# Depletion of SOD2 enhances nasopharyngeal carcinoma cell radiosensitivity via ferroptosis induction modulated by DHODH inhibition

**DOI:** 10.1186/s12885-022-10465-y

**Published:** 2023-02-03

**Authors:** Alvan Amos, Ning Jiang, Dan Zong, Jiajia Gu, Jiawei Zhou, Li Yin, Xia He, Yong Xu, Lirong Wu

**Affiliations:** 1 Department of Radiation Oncology, The Affiliated Cancer Hospital of Nanjing Medical University & Jiangsu Cancer Hospital & Jiangsu Institute of Cancer Research, 42 Baiziting Road, Nanjing, 210009 China; 2 Department of Biochemistry, Kaduna State University, PMB 2339, Tafawa Balewa Way, Kaduna, Nigeria; 3 Department of Radiation Oncology, Jiangsu Cancer Hospital & Jiangsu Institute of Cancer Research & The Affiliated Cancer Hospital of Nanjing Medical University, 42 Baiziting Road, Nanjing, 210009 China; 4 Department of Laboratory of Cancer Biology, Jiangsu Cancer Hospital & Jiangsu Institute of Cancer Research & The Affiliated Cancer Hospital of Nanjing Medical University, 42 Baiziting Road, Nanjing, 210009 China

**Keywords:** Ferroptosis, Nasopharyngeal carcinoma, Oxidative stress, SOD2, DHODH

## Abstract

**Background:**

Recurrence due to the development of radioresistance remains a major challenge in the clinical management of nasopharyngeal carcinoma. The objective of this study was to increase the sensitivity of nasopharyngeal carcinoma cells to ionizing radiation by enhancing oxidative stress and ferroptosis caused by disrupting the mitochondrial anti-oxidant enzyme system.

**Methods:**

Oxidative stress cell model was constructed by SOD2 knockdown using shRNA. The expression and activity of DHODH was suppressed by siRNA and brequinar in SOD2 depleted cells. Protein levels were determined by western blotting and ferroptosis was assessed by C11 BODIPY and malondialdehyde assay. Cell viability was evaluated using CCK-8 assay while radiotoxicity was assessed by colony formation assay. Cellular ATP level was determined by ATP assay kits, ROS was determined by DCFD and DHE, while mitochondrial oxygen consumption was determined by seahorse assay. Data were analyzed by two-tailed independent t-test.

**Results:**

Radiation upregulated SOD2 expression and SOD2 depletion increased cellular O_2_^.−^, malondialdehyde, and the fluorescence intensity of oxidized C11 BODIPY. It also resulted in mitochondrial damage. Its depletion decreased colony formation both under ionizing and non-ionizing radiation conditions. The ferroptosis inhibitor, deferoxamine, rescued cell viability and colony formation in SOD2 depleted cells. Cellular level of malondialdehyde, fluorescence intensity of oxidized C11 BODIPY, O_2_^.−^ level, ATP, and mitochondrial oxygen consumption decreased following DHODH inhibition in SOD2 depleted cells. Cell viability and colony formation was rescued by DHODH inhibition in SOD2 depleted cells.

**Conclusion:**

Inducing oxidative stress by SOD2 inhibition sensitized nasopharyngeal carcinoma cells to ionizing radiation via ferroptosis induction. This was found to be dependent on DHODH activity. This suggests that DHODH inhibitors should be used with caution during radiotherapy in nasopharyngeal carcinoma patients.

**Supplementary Information:**

The online version contains supplementary material available at 10.1186/s12885-022-10465-y.

## Background

Radiotherapy is central to the clinical management of localized tumors, one of which is nasopharyngeal carcinoma (NPC) [1]. NPC has a distinct geographical distribution with almost all cases traced to east and Southeast Asia [2]. Risk factors in NPC include Epstein-Barr virus infection, poor oral health, genetic predisposition, salted fish intake, and alcohol consumption [3]. It exhibits a high metastatic rate and more than 70% of cases are diagnosed at clinical stages III or IV [4]. Factors responsible for late diagnoses include failure to seek medical advice on time; confusing symptoms leading to wrong diagnoses; difficulty in examining the nasopharynx; and the usually normal appearance of submucosal lesions during examination of the nasopharynx [5]. In terms of demography, men are two to three times more likely to develop NPC, and peak age of occurrence is between 50 and 60 years [5]. According to GLOBOCAN 2020, there were 133, 354 new cases of cancer of the nasopharynx and 80,008 deaths worldwide [6]. World Health Organization has classified this cancer into three subtypes based on histology. These include keratinizing squamous cell carcinoma, differentiated non-keratinizing carcinoma, and undifferentiated non-keratinizing carcinoma which is the most common subtype [7]. Due to its location below the base of the skull, surgical accessibility is limited thereby making radiotherapy the primary treatment modality [7]. Development of radioresistance in NPC remains a major challenge and is the primary cause of treatment failure [8]. Therefore, it is necessary to develop effective strategies for improving its sensitivity to ionizing radiation (IR). IR produces reactive oxygen species (ROS) which can cause irreversible, lethal damage to cells by reacting with macromolecules [9]. In addition, the ROS could activate different cell death mechanisms, one of which is ferroptosis [8, 9].

Ferroptosis is a newly discovered mechanism of cell death regulation, and it is implicated in radiotoxicity [10–12]. Cells undergoing ferroptosis lack classical molecular and biochemical features of apoptotic cells but rather, they exhibit oxidative stress markers [13]. Importantly, ferroptosis is iron-dependent [13]. Studies revealed that ferroptosis is induced by extensive peroxidation of cell membrane phospholipids caused by ROS. Hence, anti-oxidant enzymes that prevent ROS accumulation or reduce peroxidized membrane phospholipids can inhibit ferroptosis. Two such enzymes are superoxide dismutase 2 (SOD2) and dihydroorotate dehydrogenase (DHODH) [14, 15]. The reaction between ROS and membrane polyunsaturated fatty acids especially arachidonic acid causes oxidative stress and activates ferroptosis [16]. Given the close association between oxidative stress and ferroptosis [17], it is conceivable that a disruption in cellular ROS homeostasis leading to ROS accumulation could induce ferroptosis and ultimately ferroptosis-mediated radiosensitivity. This disruption was achieved by the inhibition of SOD2, a mitochondrial anti-oxidant enzyme that converts O_2_^.−^ to H_2_O_2_. Hydroxyl radical is the most potent ROS involved in membrane lipids peroxidation. This radical can be produced by IR or iron-catalyzed Haber–Weiss reaction that has O_2_^.−^ as a reactant [11, 17]. DHODH, a mitochondria-localized ferroptosis inhibitor that couples de novo pyrimidine nucleotide biosynthesis to the electron transport chain (ETC), could synergize with SOD2 to inhibit ferroptosis. Therefore, DHODH was inhibited in SOD2 depleted cells to see if a synergy could be observed.

The anti-oxidant function of SOD2 is within the mitochondrial matrix [18, 19]. It converts superoxide anion (O_2_^.−^) generated by the mitochondrial ETC to H_2_O_2_. H_2_O_2_ is subsequently converted to H_2_O by catalase, peroxidases, or peroxiredoxins [20], thus protecting the cell from oxidative stress. H_2_O_2_, although a less reactive ROS compared to O_2_^.−^ and HO˙, can react with membrane polyunsaturated fatty acids or participate in the Haber–Weiss reaction to produce HO˙. A deficiency in SOD2 will result in the accumulation of O_2_^.−^ in the mitochondria, and then induce oxidative stress and ferroptosis. Recent reports confirmed the involvement of O_2_^.−^ in ferroptosis induction by erastin and doxorubicin [21, 22]. Since IR generates O_2_^.−^, SOD2 depletion could result in delayed mitochondrial O_2_^.−^ clearance and increased oxidative stress level [23]. Mitochondrial membrane lipids oxidized by ROS can be reduced by ubiquinol produced by DHODH thereby suppressing ferroptosis [15]. This enzyme links de novo pyrimidine nucleotide biosynthesis to the ETC at complex III [24]. DHODH complements mitochondrial GPX4 to reduce oxidized mitochondrial membrane phospholipids [15]. While GPX4 uses reduced glutathione (GSH) to reduce oxidized phospholipids, DHODH converts ubiquinone (CoQ10) to ubiquinol (CoQ10H_2_), which is a lipophilic anti-oxidant capable of reducing oxidized mitochondrial membrane phospholipids [15]. In this study, we evaluated the contribution of SOD2 to ferroptosis-mediated radiosensitivity in NPC cells and how this is affected by DHODH.

## Methods

### Cell culture

CNE-2 and 5-8F cell lines were obtained from the American Type Culture Collection (ATCC). The cells were periodically tested to ensure that there is no mycoplasma contamination. Cells were cultured in RPMI 1640 at 37 °C in a humidified atmosphere containing 5% CO_2._ The culture medium was supplemented with 10% (volume/volume; v/v) FBS, 1% (v/v) penicillin/streptomycin, and 1% (w/v) uridine (for cells with genetic or chemical inhibition of DHODH). Cells were cultured in 10 cm plates and seeded in 6 cm plates for fluorescence microscopy, 6-well and 12-well plates for colony formation assay (CFA), 24-well plate for the measurement of C11 BODIPY, DHE, or DCFD fluorescence intensity, and 96-well plates for cell viability assay. The cells were treated with either 5 nM siDHODH, 5 μM brequinar (DHODH inhibitor, MCE), or 5 μM DFO (ferroptosis inhibitor. Sigma, USA) as needed.

### Gene knockout/knockdown

DHODH was knocked down using siRNA (Sangon Biotech, China) and jetPRIME transfection reagent (Polyplus, China) according to the manufacturer’s guidelines. Briefly, a total of 5 nM siRNA (siDHODH, F: 5’-AUACCUGUUAAUGACAGCUUGGUCC-3’, R: 5’-GGACCAAGCUGUCAUUAACAGGUAU-3’;sictrl, F: 5’-UUCUCCGAACGUGUCACGUTT-3’, R: 5’- ACGUGACACGUUCGGAGAATT-3’), 200 µL jetprime transfection buffer and 4 µL transfection reagent were mixed and added to each well of a 6-well plate. Cells were incubated for three days. The transfection efficiency was evaluated using western blotting. SOD2 knockdown was carried out using shRNA (Sangon Biotech, China). A total of 2 μg plasmid (5′-GATCCCGGGGTTGGCTTGGTTTCAATATTCAAGAGATATTGAAACCAAGCCAACCCCTTTTTA-3′ and 5′-AGCTTAAAAAGGGGTTGGCTTGGTTTCAATATCTCTTGAATA TTGAAACCAAGCCAACCCCGG-3′), 200 μL jetprime transfection buffer and 4 μL transfection reagent were mixed and transferred to 6-well plates. Cells were incubated for three days. GPX4 KO was done using CRISPR-Cas9. A total of 2 μg plasmid (containing sgRNA and Cas9, sgGPX4: GAGATCAAAGAGTTCGCCGC [25]), 200 μL jetprime transfection buffer, and 4 μL transfection reagent were mixed and transferred to 6-well plates, and then incubated for three days_._ SOD2 knockdown and GPX4 KO cells were selected using 1 μg/mL puromycin.

### Western blotting

Western blotting for the determination of protein expression was conducted thus: cells were lysed using Beyotime Biotechnology IP lysis buffer (P0013, China). Protein concentration was determined using BCA protein assay kits (Beyotime, China). Absorbance was taken at 562 nm using a microplate spectrophotometer (Fisher Scientific, USA). Protein (10 μg) was separated on 10% SDS–polyacrylamide gel and then transferred onto nitrocellulose membranes at 200 mA for 85 min. Membranes were incubated with primary antibodies overnight at 4 °C. The antibodies used in this study were diluted as follows: SOD2 (1:1,000), GPX4 (1:1,000), DHODH (1:1,000), beta-actin (1:1,000) and GAPDH (1:1,000) (Proteintech, China). ECL detection reagent (KeyGEN, China) was used to detect the protein expression level. Images were taken using Bio-rad Gel Doc XR imaging system (Bio-Rad, USA).

### Electron microscopy

For the determination of mitochondrial damage using electron microscopy, sectioning was carried out with an ultramicrotome (Leica, EM UC7, Wetzlar, Germany). Samples were fixed with 4% paraformaldehyde and 4% glutaraldehyde in 0.1 M phosphate buffer (pH 7.4). The samples were then placed on a carbon-coated copper grid and immersed in 2% phosphotungstic acid solution for examination using JEM-1011 transmission electron microscope (JEOL Ltd., Akishima, Japan).

### Enzyme activity assay

The activity of SOD2 and GPX4 were determined using CuZn/MnSOD activity assay kit (Beyotime, China) and total glutathione peroxidase assay kit **(**Beyotime, China). The assay was performed in accordance with manufacturer’s guideline. Briefly, for SOD2 assay, cells were lysed using lysis buffer and supernatant was collected by centrifugation at 12,500 rpm for 10 min. 20 μL supernatant was added into WST-8 enzyme working solution, after which reaction working solution was added. Samples were incubated at 37 °C for 30 min. Absorbance was then determined at 450 nm. The enzyme activity was expressed as percentage inhibition of WST-8 formazan formation. To carry out total glutathione peroxidase assay, samples and the kits were prepared according to the manufacturer’s guideline. Briefly, 25 µL of samples were added to GPX detection working solution after which 10 µL of substrate (Cum-OOH) was added. Using TECAN Infinite M200 PRO microplate reader (Tecan, Switzerland), absorbance was continuously determined at 340 nm. Absorbance was taken for 7 min at 1 min interval and 25 °C. Then the enzyme activity was calculated according to kits manufacturer’s guidelines.

### Cell membrane lipids peroxidation assay

C11 BODIPY 581/591 is a widely used fluorescent sensor of membrane lipids peroxidation. To quantitatively determine the fluorescence intensity of oxidized C11-BODIPY using fluorimetry, 4 × 10^4^ cells were treated with 5 µM deferoxamine and incubated overnight. The next day, cells were incubated with 5 μM C11-BODIPY 581/591 (Invitrogen, USA) for 45 min at 37 °C. The cells were then washed three times with PBS and incubated with 5 μM Hoechst dye for 10 min. The fluorescence intensity for oxidized C11-BODIPY 581/591 and Hoechst was measured using TECAN Infinite M200 PRO microplate reader (Tecan, Switzerland). The fluorescence intensity of oxidized C11-BODIPY 581/591 was normalized to that of Hoechst. Confocal microscopy was used to qualitatively assess the level of cell membrane lipids peroxidation. 1 × 10^4^ cells were incubated with 5 µM DFO overnight. The next day, cells were treated with 5 μM C11-BODIPY 581/591 and Hoechst for 45 min and 10 min, respectively. Fluorescent images were obtained using Zeiss LSM 710 confocal microscope (ZEISS, USA). Malondialdehyde (MDA) is a biochemical marker for lipids peroxidation. Its cellular level was assessed using MDA assay kits (S0131S, Beyotime, China) according to specified guidelines. In summary, a standard curve and samples were prepared according to the manufacturer’s guidelines, after which 100 μL of samples were added to 200 μL of MDA detection working solution. The samples were then heated at 100 °C for 15 min. Heated samples were centrifuged at 5,000 rpm for 5 min and 200 μL of the supernatant was used to take absorbance at 532 nm using TECAN Infinite M200 PRO microplate reader (Tecan, Switzerland).

### ROS assay

Intracellular ROS was measured using oxidation-sensitive fluorescent probes DCFD (for total cell) and DHE (for superoxide anion). Briefly, 4 × 10^4^ cells were treated with DCFD and DHE (Invitrogen, USA). They were then incubated for 45 min and then washed, and treated with 5 μM Hoechst for 10 min. The fluorescence intensity of DHE and Hoechst was quantitatively determined using TECAN Infinite M200 PRO microplate reader (Tecan, Switzerland). The fluorescence intensity of oxidized DHE and DCFD was normalized to that of Hoechst. In order to obtain images showing the fluorescence intensities of oxidized DCFD and DHE, 4 × 10^5^ cells were incubated with DCFD and DHE for 45 min. Fluorescent images were then taken using Nikon fluorescent microscope (Nikon Instruments Inc., USA).

### Cell viability and colony formation assay

Viable cells were measured using Cell Counting Kit-8 (CCK-8, Dojindo). In summary, cells were seeded onto 96-well plates at a density of 1 × 10^4^ cells per well. 10 μL of CCK-8 reagent (in 100 μL of medium per well) were incubated for 1 h after indicated treatment and time. Absorbance was then taken at 450 nm using a microplate spectrophotometer (Fisher Scientific, USA). Cells (250 for 0 Gy or 450 for 4 Gy) were seeded on 6-well and 12-well plates and incubated for 14 days after relevant treatment. Cell colonies were dyed with methylene blue for 10 min. Pictures of adherent cells were taken using Bio-rad Gel Doc XR imaging system (Bio-Rad, USA).

### ATP assay

This assay was performed using ATP assay kits (Beyotime) according to the specified guidelines. Briefly, samples were prepared by adding ATP detection lysate solution followed by centrifugation at 12,000 rpm for 5 min. Then 20 μL of supernatant was added to 100 a μL ATP testing solution and transferred into a 96-well microplate. This was left at room temperature for 3 min. Luminescence intensity was detected using TECAN Infinite M200 PRO microplate reader (Tecan, Switzerland).

### Determination of oxygen consumption rate

Cellular oxygen consumption rate (OCR) was measured using Seahorse assay (Seahorse Bioscience, USA). 1 × 10^4^ cells were incubated in a 96-well microplate overnight. Mitochondrial oxygen consumption was determined by the sequential addition of pharmacological inhibitors of the ETC. First, baseline cellular oxygen consumption was measured and named basal oxygen consumption (BOC). 1 µM oligomycin, an inhibitor of complex V, was then added. And then, 1 µM carbonyl cyanide-ptrifluoromethoxyphenyl-hydrazone (FCCP), a protonophore, was added to collapse the inner mitochondrial membrane gradient. This drives ETC to function at its maximal capacity. Lastly, 0.5 μM antimycin A, a complex III inhibitor, and 0.5 μM rotenone, a complex I inhibitor were added to shut down the ETC.

### Statistical analysis

Data are presented as means ± standard deviation (s.d.). Statistical significance (*P* values) was calculated using unpaired Student’s t-tests. GraphPad Prism 8.0 was used for the analysis. *P < 0.05, **P < 0.01, ***P < 0.001, ****P < 0.0001, ns: not significant.

## Results

### SOD2 inhibition induced ferroptosis

**Fig. 1:**
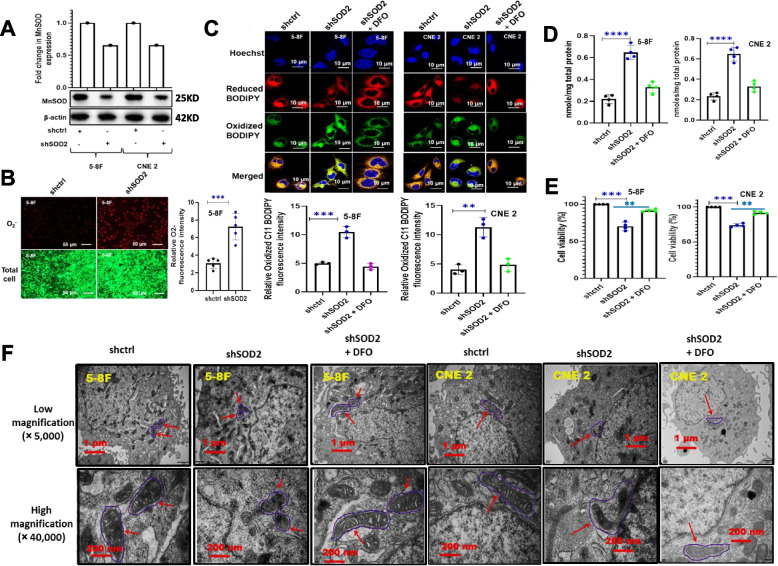
Effect of SOD2 knockdown on cellular O_2_^.−^ level and ferroptosis markers in 5-8F and CNE 2 nasopharyngeal carcinoma cells. **A** Western blot and bar plot showing MnSOD expression in control and SOD2 knockdown cells. The blots are from different parts of the same gel. **B** Fluorescence microscopy images and bar plot showing the upregulation of O_2_.^.−^ fluorescence intensity in SOD2 knockdown 5-8F cells. Cells were treated with fluorescent probes, DCFD and DHE. **C** Confocal microscopy images and bar plots showing the effect of SOD2 knockdown on oxidized C11 BODIPY 581/591 fluorescence intensity (green color). SOD2 knockdown cells were treated with 5 µM deferoxamine (DFO), an iron chelator widely used as a ferroptosis inhibitor. **D** The concentration of the lipid peroxidation marker, MDA (nmol/mg total protein). **E** Cell viability in control, SOD2 knockdown, and DFO (5 µM)-treated cells. **F** Electron micrographs showing mitochondrial damage (red arrows) in SOD2 knockdown cells which were rescued by 5 µM DFO. Mitochondrial damage is revealed by alterations in membrane structure. Data are presented as mean ± s.d. n ≥ 3 independent repeats. **P < 0.01, ***P < 0.001, ****P < 0.0001

To examine whether inhibition of SOD2 could accumulate cellular ROS and induce ferroptosis, we performed shRNA knockdown experiments in 5-8F and CNE2 cell lines followed by the determination of the cellular levels of total ROS, O_2_^.−^, MDA, and oxidized C11 BODIPY. We also carried out a cell viability assay in the presence of DFO, an iron chelator that is widely used to inhibit ferroptosis. We equally determined mitochondrial damage using electron microscopy. As shown in Fig. 1A, SOD2 knockdown was successful in both 5-8F and CNE2 cell lines. Since SOD2 is an enzyme that eliminates O_2_^.−^, SOD2 knockdown could increase O_2_^.−^ level. To determine O_2_^.−^ level, DHE, a O_2_^.−^ probe that emits red fluorescence when oxidized, was used. Our result suggested a remarkably higher O_2_^.−^ concentration in SOD2 knockdown cells (Fig. 1B). Given that O_2_^.−^ causes membrane lipids peroxidation, we conducted a lipids peroxidation assay using C11 BODIPY. The cellular level of MDA, a chemical byproduct of lipids peroxidation was also evaluated. Our result indicated a significant increase in membrane lipids peroxidation in SOD2 depleted cells as suggested by significantly higher levels of oxidized C11 BODIPY (Fig. 1C) and MDA (Fig. 1D). Oxidized C11 BODIPY and MDA level was rescued by DFO suggesting that their generation was iron-dependent. Increased membrane lipids peroxidation could cause ferroptosis, and ferroptosis could decrease cell viability. We, therefore, carried out cell viability studies in SOD2 knockdown and control cells. Our result suggested that SOD2 depleted cells had lower percentage viability which was rescued by DFO (Fig. 1E). Using electron microscopy, we evaluated the effect of SOD2 knockdown on the structural integrity of the mitochondria. As revealed in Fig. 1F, SOD2 depletion resulted in a disruption in mitochondrial structure. The result also suggested that DFO had a protective effect on the mitochondria under the SOD2 knockdown condition.

### DFO suppressed SOD2 knockdown-mediated radiosensitization

**Fig. 2:**
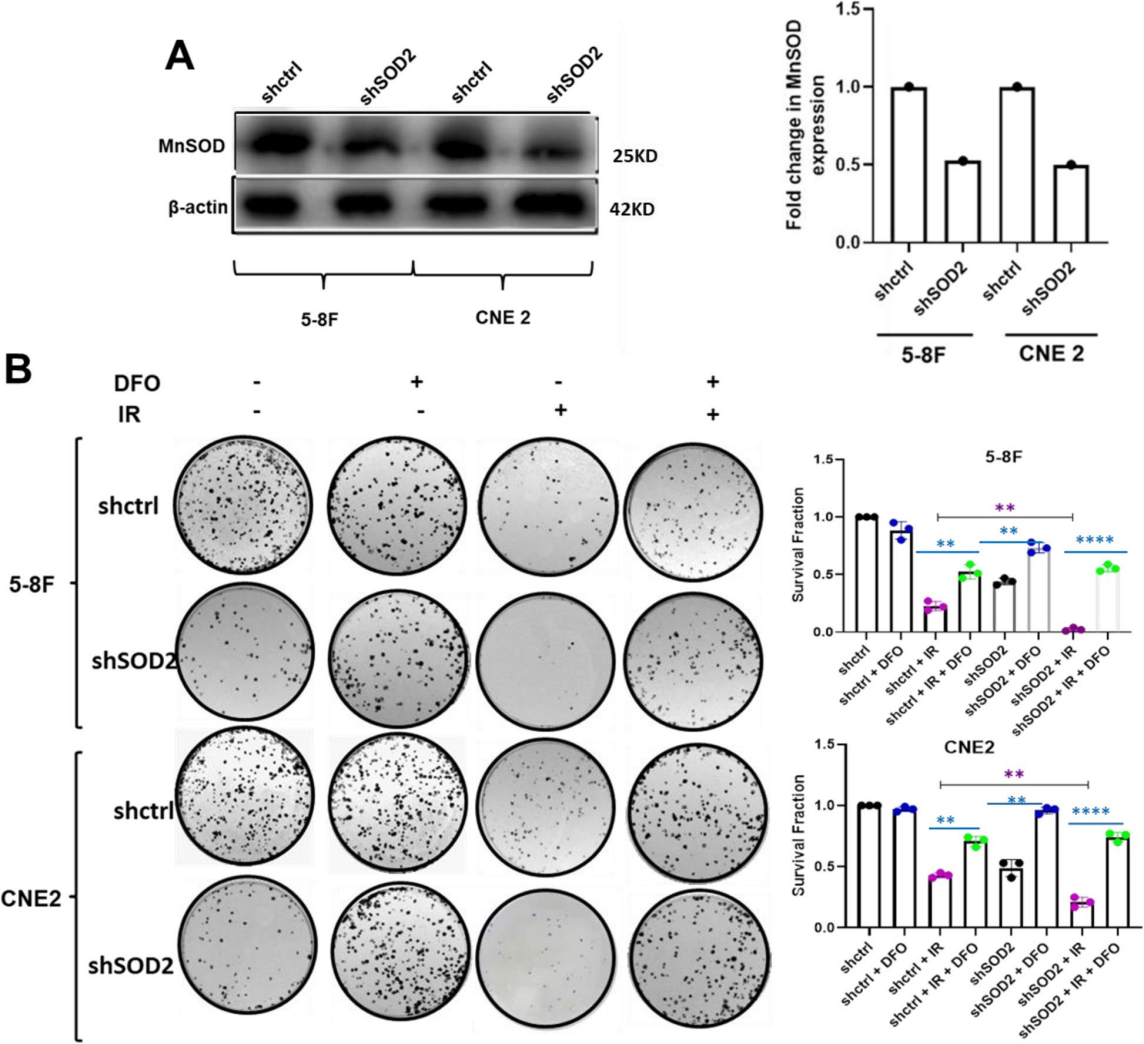
DFO inhibited SOD2 Knockdown-mediated radiosensitization. A Western blot showing the expression levels of MnSOD in 5-8F and CNE 2 cells. The blots are from different parts of the same gel. B Colony formation assay showing the effect of DFO (5 µM) on radiation-mediated cell death in SOD2 knockdown and control cells. *n* = 3 independent repeats. **P < 0.01, ****P < 0.0001. Radiation dosage: 4 Gy

Given the previous reports that SOD2 confers radioresistance to NPC cells [26], and that ferroptosis plays a key role in sensitizing cancer cells to IR [10], we carried out a colony formation assay in the presence of DFO, a ferroptosis inhibitor using SOD2 depleted and control NPC cells. This was to enable us to further ascertain whether ferroptosis could be involved in the radioresistance observed in NPC cells overexpressing SOD2 [26]. As shown in Fig. 2A, SOD2 expression was significantly reduced by shRNA-mediated knockdown in 5-8F and CNE2 cells. Consistent with the notion that SOD2 inhibits ferroptosis, knockdown of SOD2 significantly inhibited cell proliferation both under IR and non-IR conditions. Interestingly, this inhibition was markedly rescued by the treatment of SOD2 knockdown cells with DFO (Fig. 2B). These results suggest that ferroptosis is involved in the radiosensitivity and SOD2 is involved in the inhibition of radiation-mediated ferroptosis.

### DHODH inhibition suppressed ferroptosis and radiotoxicity in SOD2 depleted cells

**Fig. 3:**
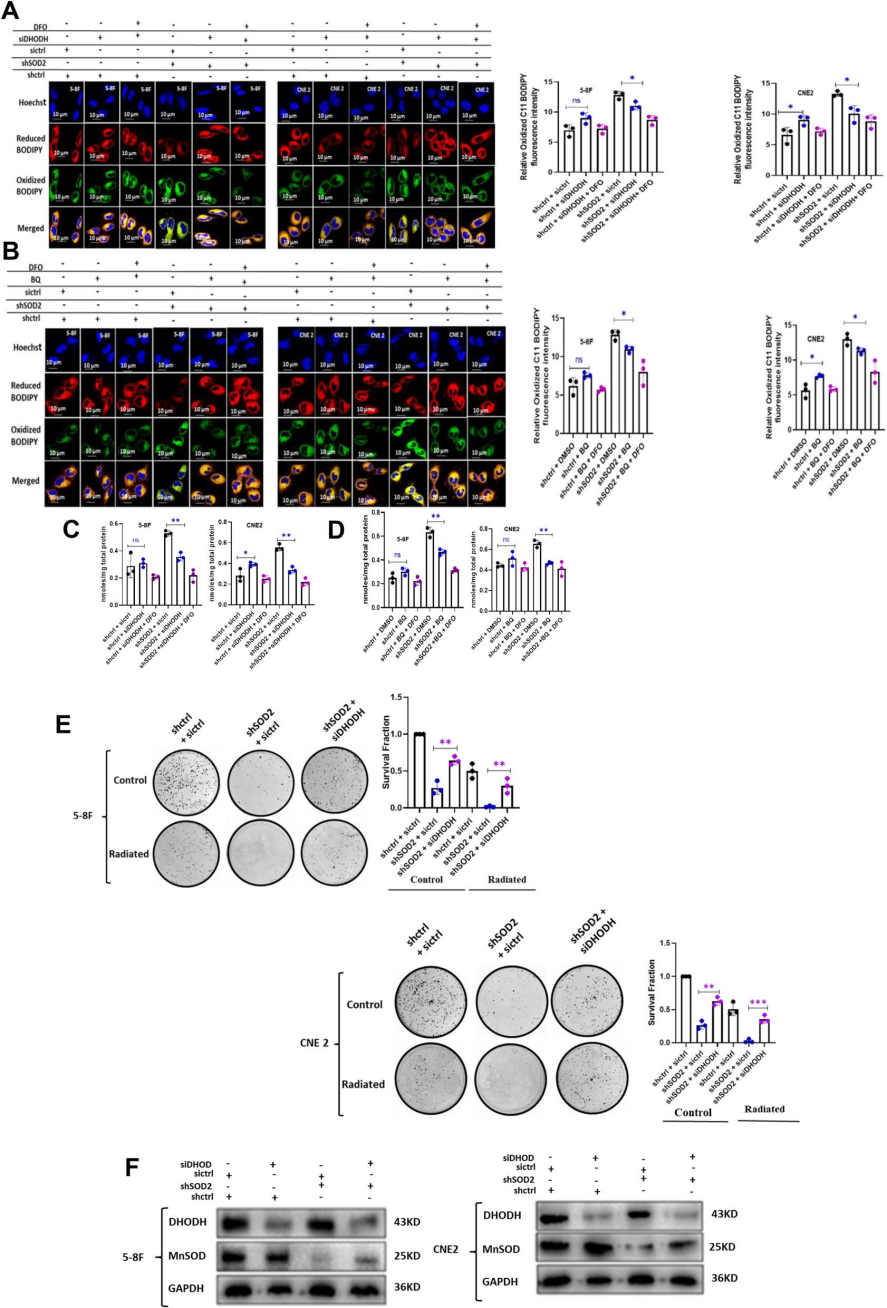
DHODH inhibition suppressed ferroptosis in SOD2 depleted cells. Confocal microscopy images and bar plots showing the fluorescence intensity of oxidized 581/591 C11 BODIPY in (**A**) SOD2 and DHODH double knockdown cells and (**B**) SOD2 knockdown cells treated with 5 µM BQ. DFO was further used to inhibit ferroptosis in DHODH inhibited and SOD2 knockdown, DHODH inhibited cells. MDA was down regulated in SOD2 depleted cells treated with (**C**) 5 nM siDHODH and (**D**) 5 µM BQ. **E** Restoration of colony formation by DHODH knockdown in SOD2 depleted 5-8F and CNE 2 cells. **F** Western blot showing DHODH and SOD2 double knockdown in 5-8F and CNE2 cells. The blots are from different parts of the same gel. *n* = 3 independent repeats. *P < 0.05; **P < 0.01. Radiation dosage: 4 Gy

Both SOD2 and DHODH are involved in the functioning of the ETC [24, 27]. SOD2 sustains ETC by eliminating O_2_^.−^ which is toxic to the mitochondria. This suggests that SOD2 depletion could inhibit ETC function. ETC is a major producer of O_2_^.−^ in the mitochondria and is involved in ferroptosis induction [28]. Previous studies suggested that complex I and II of the ETC are the main victims of SOD2 deficiency [27]. Interestingly, DHODH is linked to the ETC at complex III [24]. Therefore, it is conceivable that inhibition of complex I and II by SOD2 depletion could make DHODH be a significant contributor to O_2_^.−^ generation by the ETC. This could modulate DHODH function as a ferroptosis inhibitor. Therefore, we knocked down DHODH in SOD2 depleted cells. Our result indicated that genetic (Fig. 3A) and chemical (Fig. 3B) inhibition of DHODH slightly increased oxidized C11 BODIPY fluorescence intensity in control cells while a significant decrease was observed in SOD2 depleted cells. The slight increase is significant in CNE2 cells (p < 0.05) thus suggesting that DHODH contributes significantly to the inhibition of oxidative stress in these cell. As shown in Fig. 3C and D, DHODH inhibition in SOD2-depleted cells decreased cellular MDA levels**.** These results suggest that SOD2 inhibition may require DHODH to induce ferroptosis. We therefore examined the effect of DHODH inhibition on radiosensitization caused by SOD2 depletion. If DHODH inhibition in SOD2 knockdown cells could inhibit ferroptosis, it could reduce the radiosensitization caused by SOD2 inhibition. The result of colony formation assay suggested that siDHODH rescued colony formation following SOD2 knockdown (Fig. 3E). This suggests that DHODH may be required for sensitizing NPC cells to IR via SOD2 depletion. Western blot revealed that SOD2 and DHODH double knockdown was successful (Fig. 3F).

### DHODH inhibition reduced the level of O_2_^.−^, ATP and basal OCR after SOD2 knockdown

**Fig. 4:**
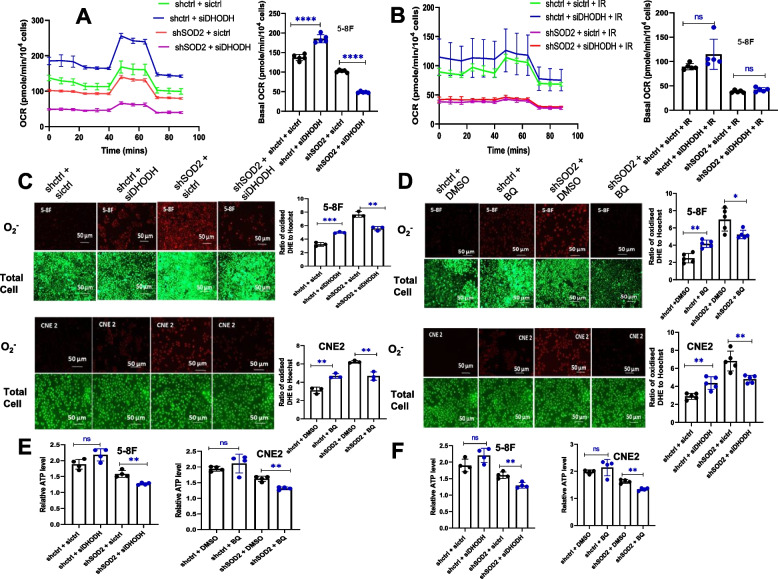
DHODH inhibition reduced the level of O_2_^.−^, ATP, and basal OCR in SOD2 knockdown cells. Effect of DHODH knockdown on OCR in SOD2 depleted and control cells (**A**) without radiation and (**B**) with radiation. Fluorescence microscopy images and bar plots showing the effect of (**C**) DHODH knockdown and (**D**) 5 µM BQ on O_2_.^−^ fluorescence intensity in control and SOD2 knockdown cells. **E** Effect of DHODH knockdown on ATP level in SOD2 depleted and control 5-8F and CNE 2 cells. **F** Effect of 5 µM BQ on ATP level in SOD2 knockdown and control 5-8F and CNE 2 cells. Radiation dosage: 4 Gy. n ≥ 3 independent repeats. *P < 0.05, **P < 0.01, ***P < 0.001

In order to establish the biochemical mechanism responsible for the effect of DHODH on ferroptosis in SOD2 depleted cells, we assessed mitochondrial function by determining OCR, ATP, and O_2_^.−^ levels in control, SOD2 knockdown and SOD2 and DHODH double knockdown cells. Our result indicated that under non-IR condition, genetic inhibition of DHODH increased basal oxygen consumption (BOC) rate in control cells while decreasing it in SOD2 knockdown cells (Fig. 4A). Under IR-conditions (Fig. 4B), the increase in BOC rate observed in control cells was not significant, and no change was observed in SOD2 depleted cells. This could be due to the effect of IR on mitochondrial function. ETC is a major producer of O_2_^.−^ in the mitochondria [29]. In addition, O_2_^.−^ production by complex III of the ETC has been linked to ferroptosis induction [28]. Our result indicated that genetic (Fig. 4C) and chemical (Fig. 4D) inhibition of DHODH significantly reduced O_2_^.−^ level in SOD2 depleted cells while increasing it in control cells. Since mitochondrial oxygen consumption is coupled to ATP synthesis, we assessed the cellular level of ATP in SOD2 knockdown and control cells treated with DHODH inhibitors. ATP level in control cells significantly increased, while DHODH inhibition resulted in a significant decrease in cellular ATP level in SOD2 knockdown cells (Fig. 4E and F). These results suggest that when SOD2 was deficient, DHODH significantly contributed to mitochondrial BOC, ATP production, and the generation of O_2_^.−^ by the ETC under non-IR conditions. Thus, when DHODH was inhibited in SOD2 depleted cells, O_2_^−^ level became reduced resulting in ferroptosis inhibition.

## Discussion

Following its first description in 2012, biochemical mechanisms that activate ferroptosis continued to emerge [13]. All reported mechanisms have oxidative stress as a common denominator [30–32]. Ferroptotic cells express high levels of oxidative stress markers [13]. Ferroptosis has recently been implicated in ionizing radiation-mediated cell death and tumor suppression [10–12]. In fact, it has been reported that ferroptosis could contribute more to radiotoxicity than apoptosis and necrosis [10]. Oxidative stress can be induced either through the endogenous generation of ROS or inhibition of anti-oxidant enzymes. Therefore, inhibition of the mitochondrial anti-oxidant enzyme, SOD2, could result in the accumulation of O_2_^.−^ [19]. This ROS plays multiple roles in the fight against cancer. In radiotherapy, O_2_^.−^ contributes to radiotoxicity [33, 34], in chemotherapy, it could be involved in drug mechanism of action [35], and in immunotherapy, O_2_^.−^ could play a role in anti-tumor immunity [36]. SOD2 converts O_2_^.−^ in the mitochondrial matrix to H_2_O_2_, which is further converted to water by catalase, peroxiredoxins, or thioredoxins [19, 37]. Given that IR could generate ROS (including O_2_^.−^) in cells [33, 34], SOD2 depletion could result in further O_2_^.−^ accumulation in the mitochondria. This is because, the mitochondria will not be able to get rid of the O_2_^.−^ generated by the IR. Therefore, mitochondrial oxidative stress caused by SOD2 depletion could facilitate radiation-mediated ferroptosis and enhance the sensitivity of NPC cells to IR [21, 28]. Ferroptosis-related signaling pathways can be categorized into (i) those related to intracellular iron homeostasis, (ii) those related to cell membrane damage/repair and lipids metabolism, and (iii) those related to the homeostatic regulation of ROS. Radiation-mediated modulation in the expression of proteins related to these pathways could affect ferroptosis.

Excessive accumulation of O_2_^.−^ caused by SOD2 inhibition resulted in enhanced membrane lipids peroxidation and ferroptosis. This was observed both under IR and non-IR conditions. Our finding agrees with a previous report, which revealed that endogenous generation of O_2_^.−^ by sodium selenite is pro-ferroptotic [38]. In addition, decreased cellular anti-oxidant capacity has been shown to induce ferroptosis [39]. The ferroptosis inhibitor, DFO, could reverse the increase in lipid peroxidation caused by SOD2 depletion. Indeed, DFO treatment restored cell growth inhibition by SOD2 knockdown (Figs. 1E and 2B). This suggests the involvement of ferroptosis in suppressing cell survival in response to SOD2 knockdown. In agreement with previous reports, data from our CFA has indicated a significant increase in radiosensitivity in response to SOD2 knockdown. Our result indicated that SOD2 depletion induces ferroptosis while previous reports revealed that ferroptosis enhances radiosensitivity [10, 40]. It is worth noting that IR could induce epithelial to mesenchymal transition (EMT), cancer cells’ biochemical transformation that precedes metastasis. Although cells that have assumed the mesenchymal phenotype have been reported to be radioresistant [41, 42], EMT promotes the susceptibility of head and neck cancers to ferroptosis ^47^. This suggests that sensitizing nasopharyngeal carcinoma cells to ferroptosis using inducers such as erastin, RSL3 or SOD2 inhibitors could be a viable strategy for inhibiting metastasis facilitated by IR. NPC has the highest metastatic potential among all head and neck cancers, [43, 44] therefore; this anti-metastasis strategy is worth exploring.

It has been reported that mitochondria play an important role in ferroptosis [21, 22, 28]. Given the key role played by SOD2 and DHODH in mitochondrial function, as well as ferroptosis, we carried out chemical and genetic inhibition of DHODH in control and SOD2 knockdown NPC cells. This was to find out whether DHODH will modulate ferroptosis induced by SOD2 depletion. Our result suggests that DHODH may be required to sensitize NPC cells to radiation via ferroptosis induction caused by SOD2 inhibition. The significant decrease in OCR, O_2_^.−^, and ATP observed when DHODH was inhibited in SOD2 depleted cells suggests that SOD2 depletion rewired mitochondrial metabolism in such a way that made DHODH to play a significant role in ETC function. This is because the ETC plays a key role in oxygen consumption, ATP, and O_2_^.−^ generation [28, 45]. Unexpectedly, there was no significant change in basal oxygen consumption under IR conditions. This could be due to the multiple signaling pathways activated by radiation, which could affect mitochondrial function. Diminished ferroptosis and radiosensitivity observed under DHODH and SOD2 double knockdown conditions could be due to the failure of the mitochondria to sufficiently produce O_2_^.−^ and ATP [28, 45]. This conclusion is in agreement with the role of O_2_^.−^ in oxidative stress and ferroptosis. It also agrees with a recent report which revealed that energy stress caused by ATP depletion could inhibit ferroptosis through the activation of AMP-dependent protein kinase [45].

Previous studies revealed that oxidative stress reprograms cancer cell metabolism to upregulate glycolysis while downregulating the mitochondria-localized tricarboxylic acid (TCA) cycle and the ETC [27]. This metabolic rewiring called the Warburg effect could be an adaptive response activated to regulate cellular ROS level [27]. This is because mitochondrial ETC is the primary source of O_2_^.−^ [27], so the cell shuts it down via activating the Warburg effect when cellular ROS goes above a certain threshold level. However, shutting down complex III of the ETC by this metabolic reprogramming could inhibit DHODH-catalyzed reaction which is the rate- limiting step in de novo pyrimidine nucleotide biosynthesis. This reaction is coupled to the ETC at complex III and is required for cancer cell proliferation [46]. It is therefore conceivable that the Warburg effect only affects complex I and II of the ETC. SOD2 deficiency has been reported to induce the Warburg effect [27, 47]. Therefore, it is likely that SOD2 depletion inhibited only complex I and II of the ETC thus making the ETC to be dependent on DHODH-catalyzed reaction.

**Fig. 5:**
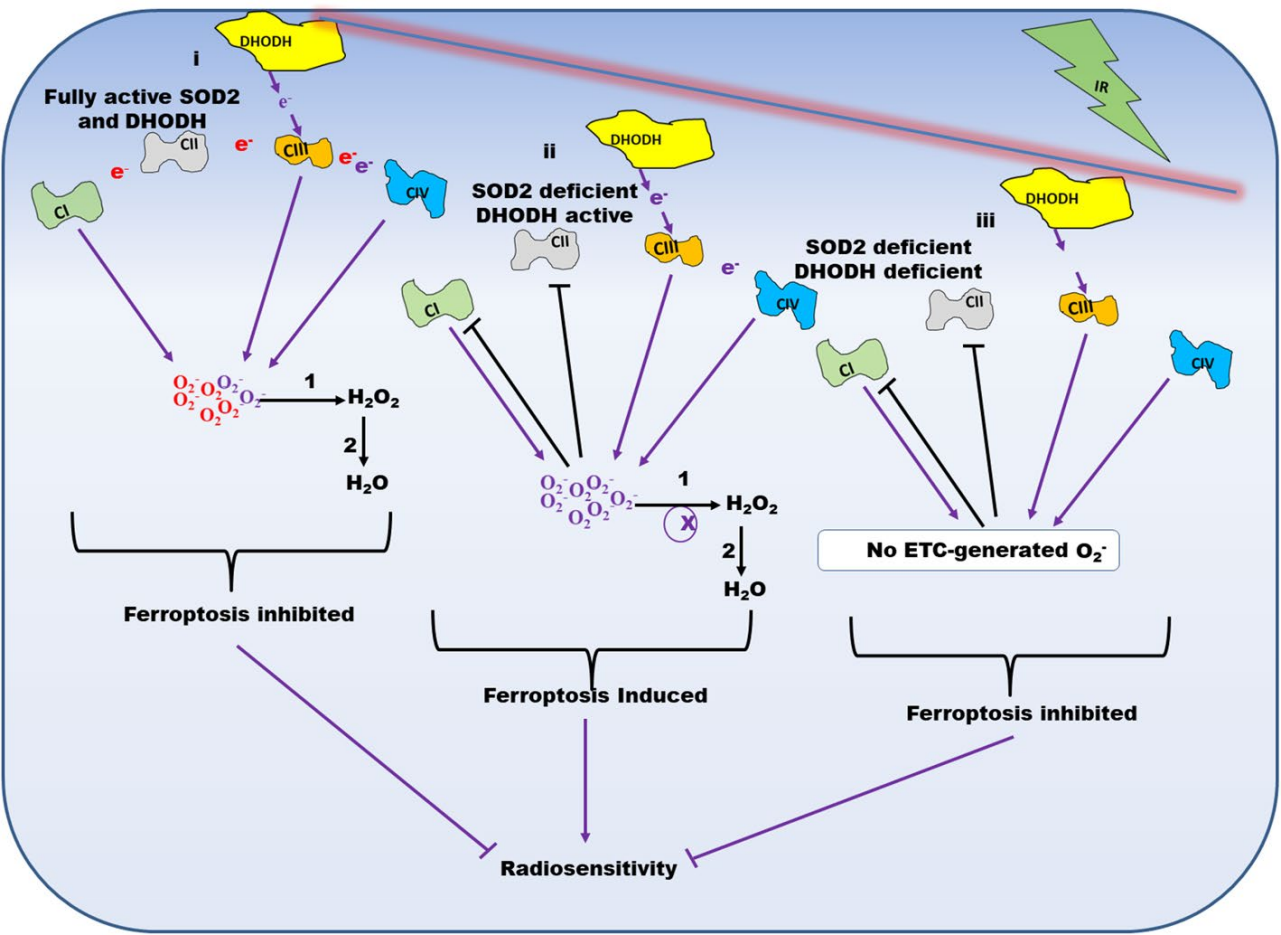
Schematic representation of the effect of DHODH knockdown on ferroptosis-mediated radiosensitivity caused by SOD2 depletion. When both SOD2 and DHODH are fully active, Ci and Ciii of the ETC generate O_2_^.−^. SOD2 converts the O_2_^.−^ to H_2_O_2_ which is converted to H_2_O by catalase, peroxidases, or thioredoxins. This results in ferroptosis inhibition. When only SOD2 is deficient, the Warburg effect inhibits Ci and Cii, hence only Ciii generate O_2_^.−^. Under this condition, O_2_^.−^ accumulates and ferroptosis is induced thereby enhancing radiosensitivity. When both SOD2 and DHODH are depleted, the Warburg effect inhibits Ci and Cii, and DHODH does not facilitate the generation of O_2_^.−^ by Ciii. Therefore, no O_2_^.−^ is produced by the ETC. This inhibits ferroptosis and radiosensitivity. *Ci = Complex I; Cii = Complex II; Ciii = Complex = III; Civ = Complex IV; 1 = SOD2; 2 = Catalase, Peroxidases or Thioredoxins; ETC = Electron Transport Chain

Inhibition of complex I and II of the ETC by the Warburg effect was previously reported [47]. Since complex I and III are the producers of O_2_^.−^ in the ETC, shutting down complex I through the Warburg effect will make O_2_^.−^ generation by the ETC dependent on complex III. Our result suggests that under SOD2 depleted conditions, the coupling of DHODH-catalyzed reaction to complex III facilitates oxygen consumption, ATP, and O_2_^.−^ generation. Interestingly, O_2_^.−^ released by complex III of the ETC has been reported to be responsible for cysteine deprivation-induced ferroptosis [28]. This is likely due to the ability of complex III to release O_2_^.−^ both into the mitochondrial matrix and intermembrane space unlike complex I which only releases O_2_^.−^ into the mitochondrial matrix [28, 48]. It was reported that inhibitors of the ETC that targets either complex I or II could not sustainably inhibit ferroptosis while complex III and IV inhibitors were able to sustainably inhibit it [21]. Pharmacological instability of the complex I and II inhibitors was advanced as the possible reason. However, our result suggests that sustenance of the ETC by DHODH-catalyzed reaction could be an alternative explanation. A schematic representation of the effect of DHODH depletion on ferroptosis induced by SOD2 inhibition is shown in Fig. 5.

Since solid tumors are characterized by oxidative stress and the Warburg effect [49, 50], the biochemical phenotype of these tumors could resemble that of our oxidative stress cell model. Therefore, pharmacological agents that target DHODH could inhibit ferroptosis resulting in diminished efficacy or radiosensitivity. Studying the effect of DHODH inhibition on ferroptosis in solid tumors is therefore recommended. Given that oxidation of membrane phospholipids especially polyunsaturated fatty acids (PUFA) is the main driver of ferroptosis, tumors originating from tissues rich in membrane PUFA may be highly susceptible to ferroptosis. However, through metabolic rewiring at the initial stage of tumorigenesis, tumors could alter their PUFA composition to evade ferroptosis. Whether this occurs, could be an interesting area to explore.

## Conclusion

This study revealed that disrupting ROS homeostasis by SOD2 depletion could induce ferroptosis via accumulating O_2_^.−^ thereby sensitizing NPC cells to IR. Also, DHODH inhibition could suppress ferroptosis induced by SOD2 depletion. This implies that care should be taken when using DHODH inhibitors in NPC patients undergoing radiotherapy.

## Supplementary Information

**Additional file 1. **Blots were cut prior to hybridization withantibodies.

## Data Availability

The data generated during the study are available from the corresponding author on reasonable request.

## Abbreviations

SOD2: Superoxide Dismutase 2
DHODH: Dihydroorotate Dehydrogenase
shRNA: Short Hairpin Ribonucleic Acid
siRNA: Small Interfering Ribonucleic Acid
BODIPY: Boron Dipyrromethene Difluoride
CCK-8: Cell Counting Kit-8
ATP: Adenosine Triphosphate
ROS: Reactive Oxygen Species
DHE: Dihydroethidium
GLOBOCAN: Global Cancer Statistics
NPC: Nasopharyngeal Carcinoma
IR: Ionizing Radiation
ETC: Electron Transport Chain
GPX4: Glutathione Peroxidase 4
GSH: Reduced Glutathione
CoQ10: Ubiquinone
CoQ10H_2_: Ubiquinol
ATCC: American Type Culture Collection
RPMI: Rosewell Park Memorial Institute
FBS: Fetal Bovine Serum
CFA: Colony Formation Assay
DFO: Deferoxamine
KO: Knockout
CuZn/MnSOD: Copper Zinc/Manganese Superoxide Dismutase
WST-8: Water-soluble Tetrazolium Dye-8
MDA: Malondialdehyde
OCR: Oxygen Consumption Rate
BOC: Basal Oxygen Consumption
FCCP: Carbonyl Cyanide-Ptrifluoromethoxyphenyl-Hydrazone
