# Correction: Depletion of SOD2 enhances nasopharyngeal carcinoma cell radiosensitivity via ferroptosis induction modulated by DHODH inhibition

**DOI:** 10.1186/s12885-023-10969-1

**Published:** 2023-05-19

**Authors:** Alvan Amos, Ning Jiang, Dan Zong, Jiajia Gu, Jiawei Zhou, Li Yin, Xia He, Yong Xu, Lirong Wu

**Affiliations:** 1 Department of Radiation Oncology, The Affiliated Cancer Hospital of Nanjing Medical University & Jiangsu Cancer Hospital & Jiangsu Institute of Cancer Research, 42 Baiziting Road, Nanjing, 210009 China; 2 Department of Biochemistry, Kaduna State University, PMB 2339, Tafawa Balewa Way, Kaduna, Nigeria; 3 Department of Radiation Oncology, Jiangsu Cancer Hospital & Jiangsu Institute of Cancer Research & The Affiliated Cancer Hospital of Nanjing Medical University, 42 Baiziting Road, Nanjing, 210009 China; 4 Department of Laboratory of Cancer Biology, Jiangsu Cancer Hospital & Jiangsu Institute of Cancer Research & The Affiliated Cancer Hospital of Nanjing Medical University, 42 Baiziting Road, Nanjing, 210009 China

**Correction: BMC Cancer 23, 117 (2023)**

10.1186/s12885-022-10465-y

Following publication of the original article [1], the authors identified a typesetting error whereby the corresponding authors were not correctly marked. The correct corresponding authors are:

Xia He, hexiabm@163.com; Yong Xu, yxu4696@njmu.edu.cn; Lirong Wu, wulirong126@126.com

The author group in this correction article has been updated and the original article [1] has been corrected.

## References

[1] Amos A, Jiang N, Zong D (2023). Depletion of SOD2 enhances nasopharyngeal carcinoma cell radiosensitivity via ferroptosis induction modulated by DHODH inhibition. BMC Cancer.

