# Supplementary material for: Depletion of SOD2 enhances nasopharyngeal carcinoma cell radiosensitivity via ferroptosis induction modulated by DHODH inhibition
Source: BMC Cancer. 2023 Feb 3;23:117. doi: 10.1186/s12885-022-10465-y (PMC9896811; doi:10.1186/s12885-022-10465-y)

Original blots

**Depletion of SOD2 Enhances Nasopharyngeal Carcinoma Cell Radiosensitivity via Ferroptosis Induction Modulated by DHODH Inhibition**

Alvan Amos**^1,2^**, Ning Jiang**^3^**, Dan Zong**^3^**, Jiajia Gu**^3^**, Jiawei Zhou^1^, Li Yin**^3^**, Xia He**^3^**, Yong Xu**^4^**, Lirong Wu**^3^**

**Blots were cut prior to hybridization with antibodies**

**Figure 1A**

**
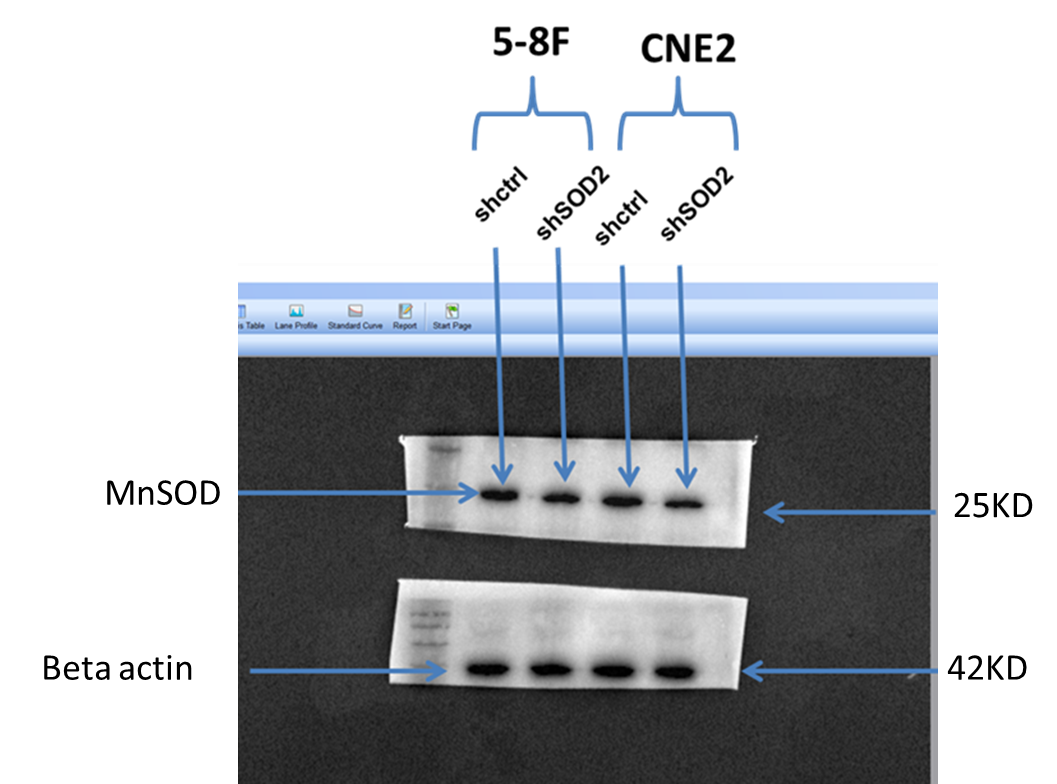
**

**Replicate**

**
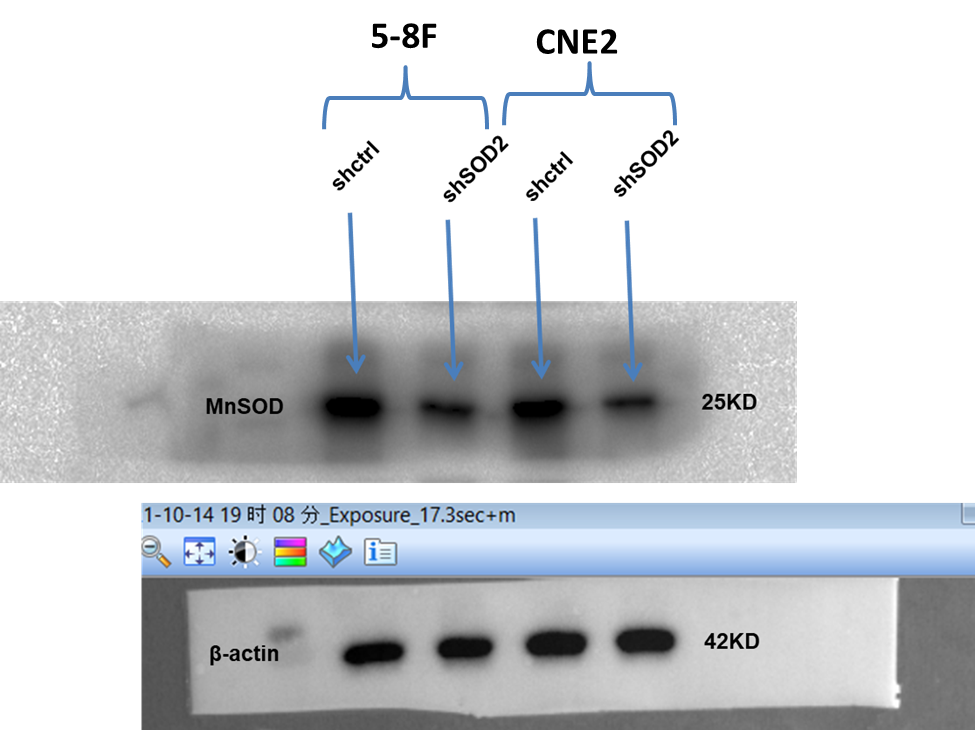
**

**Figure 2A**

**
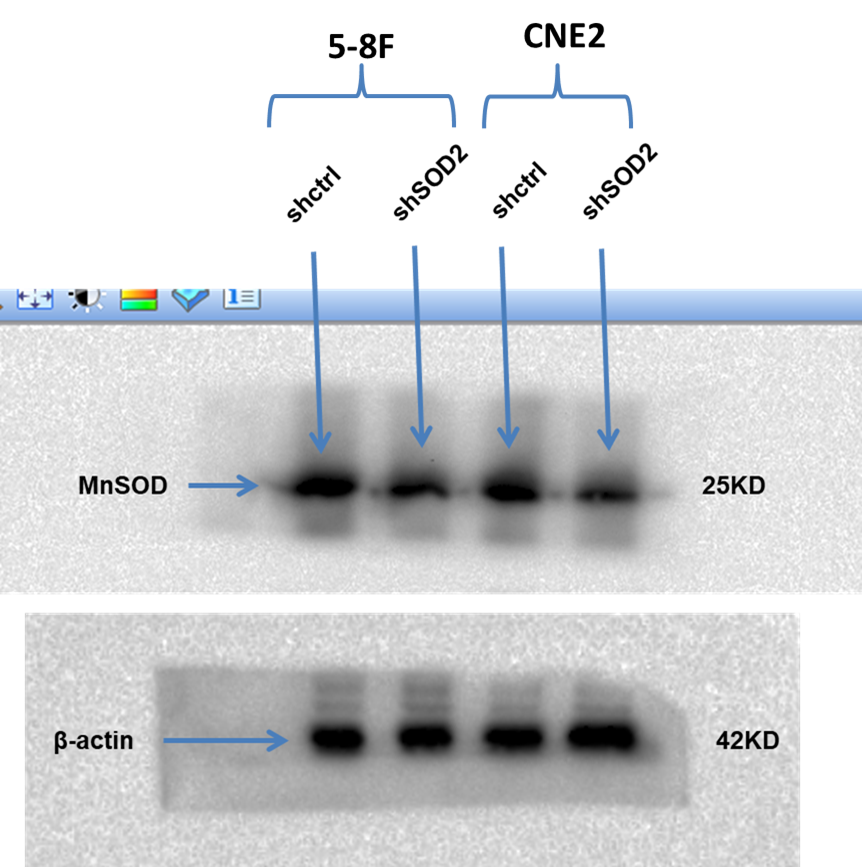
**

**Replicate**


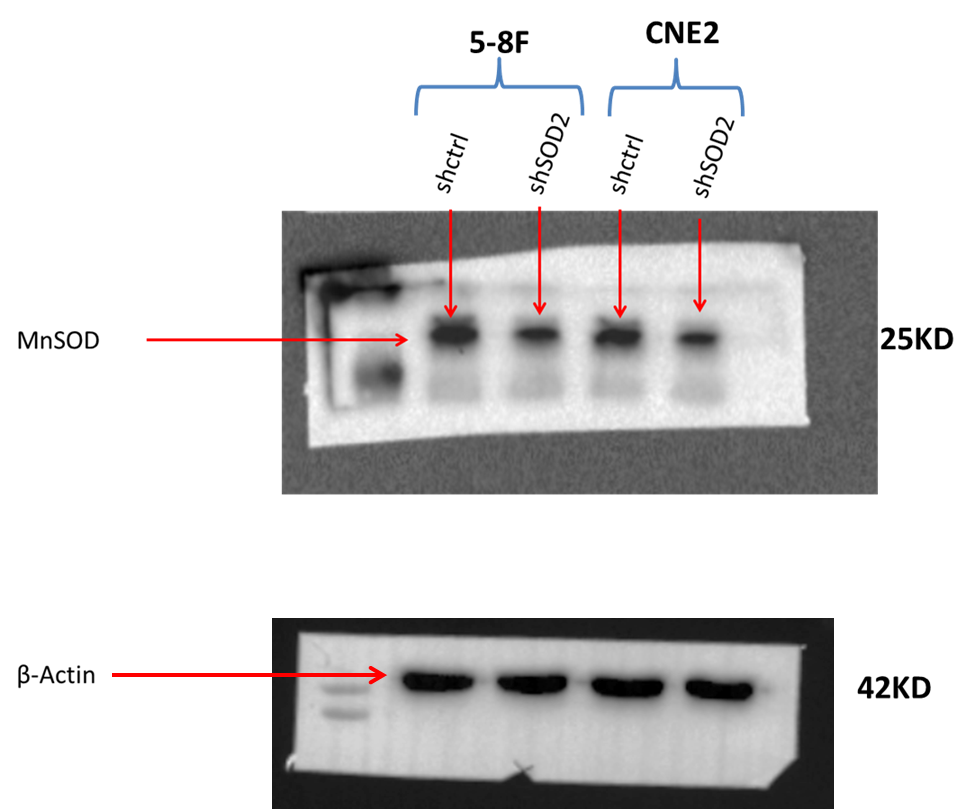


**Figure 3F**


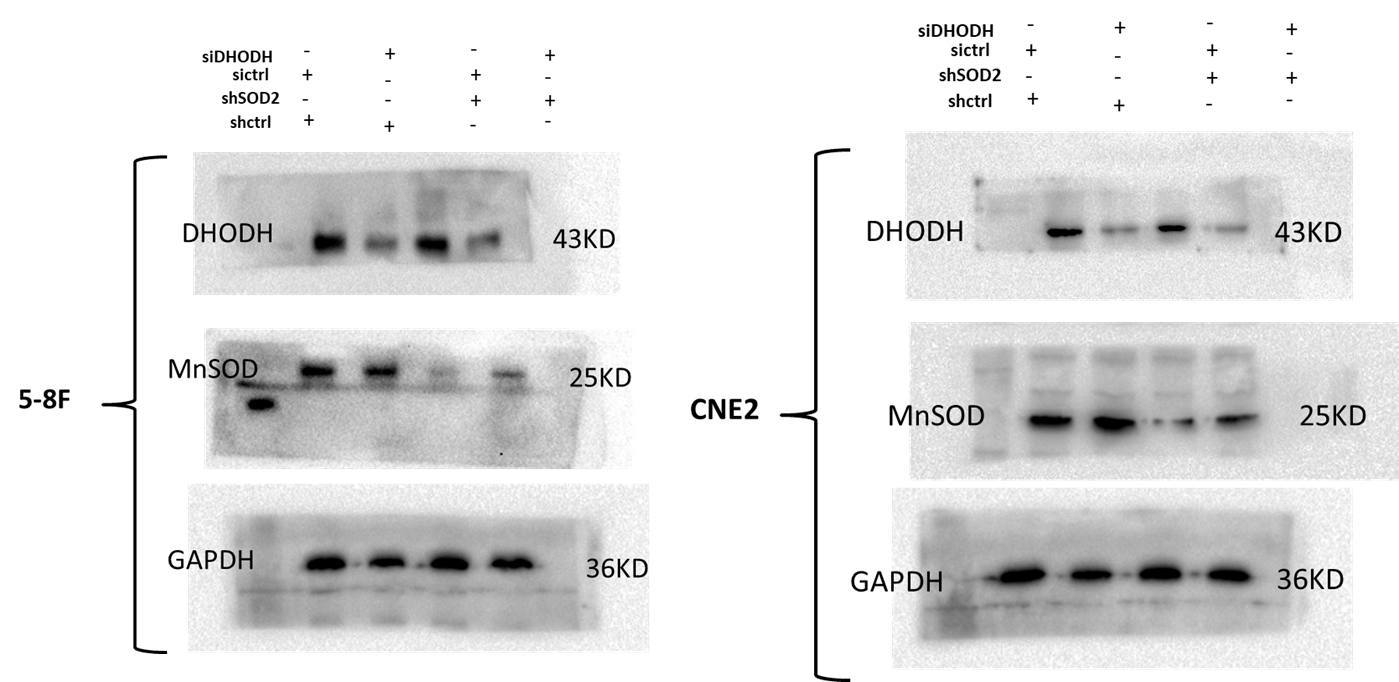


**Replicate**


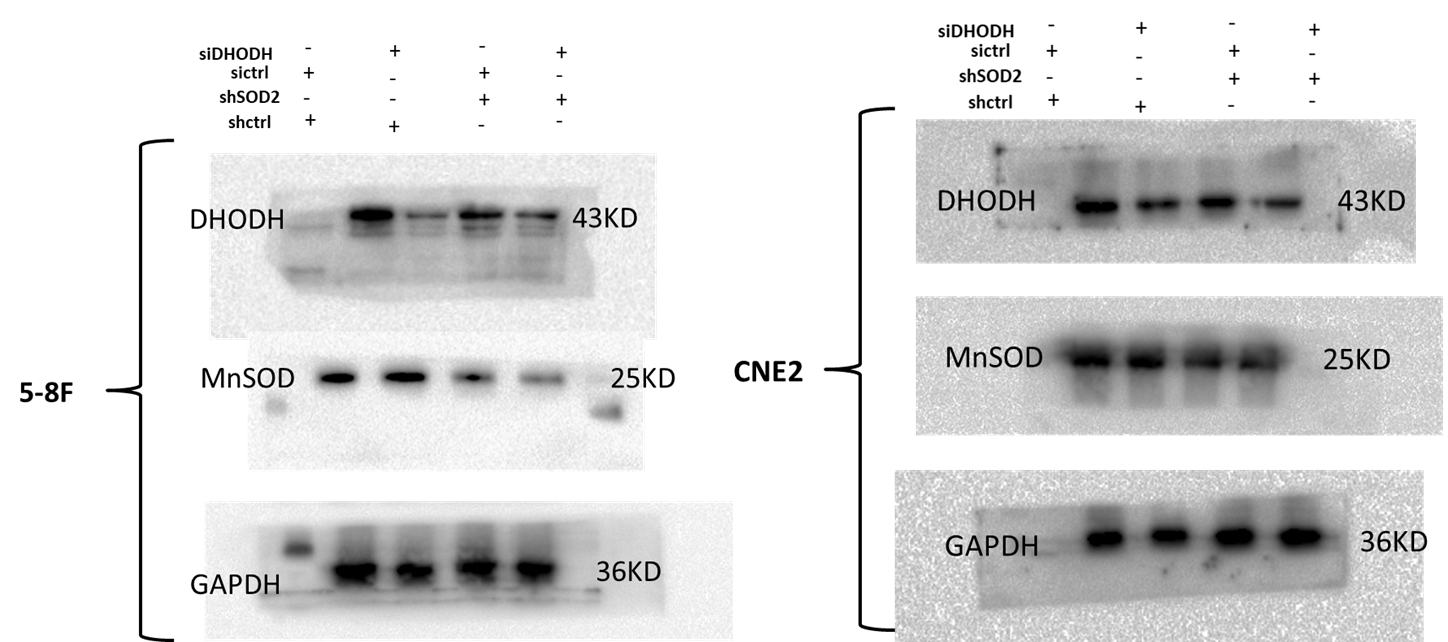

Supplement: Supplementary file 1 — Additional file 1. Blots were cut prior to hybridization withantibodies. [file 12885_2022_10465_MOESM1_ESM.docx]
